# Nonnegative Matrix Factorization with Gaussian Process Priors

**DOI:** 10.1155/2008/361705

**Published:** 2008-04-21

**Authors:** Mikkel N. Schmidt, Hans Laurberg

**Affiliations:** ^1^Department of Informatics and Mathematical Modelling, Technical University of Denmark, Richard Petersens Plads, DTU-Building 321, 2800 Lyngby, Denmark; ^2^Department of Electronic Systems, Aalborg University, Niels Jernes Vej 12, 9220 Aalborg Ø., Denmark

## Abstract

We present a general method for including prior knowledge in a nonnegative matrix factorization (NMF), based on Gaussian process priors. We assume that the nonnegative factors in the NMF are linked by a strictly increasing function to an underlying Gaussian process specified by its covariance function. This allows us to find NMF decompositions that agree with our prior knowledge of the distribution of the factors, such as sparseness, smoothness, and symmetries. The method is demonstrated with an example from chemical shift brain imaging.

## 1. Introduction

Nonnegative matrix factorization (NMF) [1, 2] is a recent method for factorizing a matrix as the product of two matrices, in which all elements are nonnegative. NMF has found widespread application in many different areas including pattern recognition [3], clustering [4], dimensionality reduction [5], and spectral analysis [6, 7]. Many physical signals, such as pixel intensities, amplitude spectra, and occurrence counts, are naturally represented by nonnegative numbers. In the analysis of mixtures of such data, nonnegativity of the individual components is a reasonable constraint. Recently, a very simple algorithm [8] for computing the NMF was introduced. This has initiated much research aimed at developing more robust and efficient algorithms.

Efforts have been made to enhance the quality of the NMF by adding further constraints to the decomposition, such as sparsity [9], spatial localization [10, 11], and smoothness [11, 12], or by extending the model to be convolutive [13, 14]. Many extended NMF methods are derived by adding appropriate constraints and penalty terms to a cost function. Alternatively, NMF methods can be derived in a probabilistic setting, based on the distribution of the data [6, 15–17]. These approaches have the advantage that the underlying assumptions in the model are made explicit.

In this paper, we present a general method for using prior knowledge to improve the quality of the solutions in NMF. The method is derived in a probabilistic setting, and it is based on defining prior probability distributions of the factors in the NMF model in a Gaussian process framework. We assume that the nonnegative factors in the NMF are linked by a strictly increasing function to an underlying Gaussian process, specified by its covariance function. By specifying the covariance of the underlying process, we can compute NMF decompositions that agree with our prior knowledge of the factors, such as sparseness, smoothness, and symmetries. We refer to the proposed method as nonnegative matrix factorization with Gaussian process priors, or GPP-NMF for short.

## 2. NMF with Gaussian Process Priors

In the following, we derive a method for including prior information in an NMF decomposition by assuming Gaussian process priors (for a general introduction to Gaussian processes, see, e.g., Rasmussen and Williams [18].) In our approach, the Gaussian process priors are linked to the nonnegative factors in the NMF by a suitable link function. To setup the notation, we start by deriving the standard NMF method as a maximum likelihood (ML) estimator and then move on to the maximum a posteriori (MAP) estimator. Then, we discuss Gaussian process priors and introduce a change of variable that gives better optimization properties. Finally, we discuss the selection of the link function.

### 2.1. Maximum Likelihood NMF

The NMF problem can be stated as

(1) X=DH+N,

where **X** ∈ ℝ^*K*×*L*^ is a data matrix that is factorized as the product of two element-wise nonnegative matrices, **D** ∈ ℝ_+_^*K*×*M*^ and **H** ∈ ℝ_+_^*M*×*L*^, where ℝ_+_ denotes the nonnegative reals. The matrix **N** ∈ ℝ^*K*×*L*^ is the residual noise.

There exists a number of different algorithms [8, 15–17, 19–21] for computing this factorization, some of which can be viewed as maximum likelihood methods under certain assumptions about the distribution of the data. For example, least squares NMF corresponds to i.i.d. Gaussian noise [17], and Kullback-Leibler NMF corresponds to a Poisson process [6].

The ML estimate of **D** and **H** is given by

(2) $$\{D_{\text{ML}},H_{\text{ML}}\}=\underset{D,H\geq 0}{\arg\min}{ℒ}_{X|D,H}(D,H),$$

where *ℒ*_*X*∣*D*,*H*_(**D**, **H**) is the negative log likelihood of the factors.

**Example 1 (least squares NMF).**

An example of a maximum likelihood NMF is the least squares estimate. If the noise is i.i.d. Gaussian with variance *σ_N_* ^2^, the likelihood of the factors **D** and **H** can be written as

(3) $$p_{X|D,H}^{\text{LS}}(X\;|\;D,H)=\frac{1}{{\left(\sqrt{2\pi }\sigma_{N}\right)}^{KL}}\exp\left(-\frac{∥X-DH{∥}_{F}^{2}}{2\sigma_{N}^{2}}\right).$$

The negative log likelihood, which serves as a cost function for optimization, is then

(4) $${ℒ}_{X|D,H}^{\text{LS}}(D,H)\propto \frac{1}{2\sigma_{N}^{2}}∥X-DH{∥}_{F}^{2},$$

where we use the proportionality symbol to denote equality subject to an additive constant. To compute a maximum likelihood estimate of **D** and **H**, the gradient of the negative log likelihood is useful:

(5) $$\nabla_{H}{ℒ}_{X|D,H}^{\text{LS}}(D,H)=\frac{1}{\sigma_{N}^{2}}D^{⊤}(DH-X),$$

and the gradient with respect to **D**, which is easy to derive, is similar because of the symmetry of the NMF problem.

The ML estimate can be computed by multiplicative update rules based on the gradient [8], projected gradient descent [19], alternating least squares [20], Newton-type methods [21], or any other appropriate constrained optimization method.

### 2.2. Maximum a Posteriori NMF

In this paper, we propose a method to build prior knowledge into the solution of the NMF problem. We choose a prior distribution *p_D,H_*(**D**, **H**) over the factors in the model, that captures our prior beliefs and uncertainties of the solution we seek. We then compute the maximum a posteriori (MAP) estimate of the factors. Using Bayes' rule, the posterior is given by

(6) $$p_{D,H|X}(D,H\;|\;X)=\frac{p_{X|D,H}(X\;|\;D,H)p_{D,H}(D,H)}{p_{X}(X)}.$$

Since the numerator is constant, the negative log posterior is the sum of a likelihood term that penalizes model misfit, and a prior term that penalizes solutions that are unlikely under the prior:

(7) $${ℒ}_{D,H|X}(D,H)\propto {ℒ}_{X|D,H}(D,H)+{ℒ}_{D,H}(D,H).$$

The MAP estimate of **D** and **H** is

(8) $$\{D_{\text{MAP}},H_{\text{MAP}}\}=\underset{D,H\geq 0}{\arg\min}{ℒ}_{D,H|X}(D,H),$$

and it can again be computed using any appropriate optimization algorithm.

**Example 2 (nonnegative sparse coding).**

An example of a MAP NMF is nonnegative sparse coding (NNSC) [9, 22], where the prior on **H** is i.i.d. exponential, and the prior on **D** is flat with each column constrained to have unit norm

(9) $$p_{D,H}^{\text{NNSC}}(D,H)=\underset{i,j}{\prod }\lambda \exp\left(-\lambda H_{i,j}\right),\;\;∥D_{k}∥=1\;\;\forall k,$$

where ||**D** *_k_*|| is the Euclidean norm of the *k*th column of **D**. This corresponds to the following penalty term in the cost function:

(10) $${ℒ}_{D,H}^{\text{NNSC}}(D,H)\propto \lambda \sum_{i,j}H_{i,j}.$$

The gradient of the negative log prior with respect to **H** is then

(11) $$\nabla_{H}{ℒ}_{D,H}^{\text{NNSC}}=\lambda ,$$

and the gradient with respect to **D** is zero, with the further normalization constraint given in (9).

### 2.3. Gaussian Process Priors

In the following, we derive the MAP estimate under the assumption that the nonnegative matrices **D** and **H** are independently determined by a Gaussian process [18] connected by a link function. The Gaussian process framework provides a principled and practical approach to the specification of the prior probability distribution of the factors in the NMF model. The prior is specified in terms of two functions: (i) a covariance function that describes corellations in the factors and (ii) a link function, that transforms the Gaussian process prior into a desired distribution over the nonnegative reals.

We assume that **D** and **H** are independent, so that we may write

(12) $${ℒ}_{D,H}(D,H)={ℒ}_{D}(D)+{ℒ}_{H}(H).$$

In the following, we consider only the prior for **H**, since the treatment of **D** is equivalent due to the symmetry of the NMF problem. We assume that there is an underlying variable vector, **h** ∈ ℝ^*LM*^, which is zero-mean multivariate Gaussian with covariance matrix **Σ**_*h*_:

(13) $$p_{h}(h)={\left(2\pi |\Sigma_{h}{|}^{2}\right)}^{-(1/2)NL}\text{exp}\left(-\frac{1}{2}h^{⊤}\Sigma_{h}^{-1}h\right),$$

and linked to **H** via a link function, *f_h_*: ℝ_+_ → ℝ as

(14) $$h=f_{h}(\text{vec}\left(H\right)),$$

which operates element-wise on its input. The vec (⋅) function in the expression stacks its matrix operand column by column. The link function should be strictly increasing, which ensures that the inverse exists. Later, we will further assume that the derivatives of *f_h_* and *f_h_* ^−1^ exist. Under these assumptions, the prior over **H** is given by (using the change of variables theorem)

(15) $$\begin{array}{ll} p_{H}(H) & =p_{h}(f_{h}(\text{vec}\left(H\right)))\;|\;J(f_{h}(\text{vec}\left(H\right)))\;|\\ & \propto \text{exp}(-\frac{1}{2}f_{h}(\text{vec}\left(H\right){)}^{⊤}\Sigma_{h}^{-1}f_{h}(\text{vec}\left(H\right)))\underset{i}{\prod }\;|\;f_{h}^{\prime }(\text{vec}\left(H\right){)\;|\;}_{i}, \end{array}$$

where *𝒥*(⋅) denotes the Jacobian determinant and *f_h_*′ is the derivative of the link function. The negative log prior is then

(16) $${ℒ}_{H}(H)\propto \frac{1}{2}f_{h}(\text{vec}\left(H\right){)}^{⊤}\Sigma_{h}^{-1}f_{h}(\text{vec}\left(H\right))-\underset{i}{\sum }\log\;|\;f_{h}^{\prime }(\text{vec}\left(H\right){)\;|\;}_{i}.$$

This expression can be combined with an appropriate likelihood function, such as the least-squares likelihood in (4) and can be optimized to yield the MAP solution; however, in our experiments, we found that a more simple and robust algorithm can be obtained by making a change of variable as explained next.

### 2.4. Change of Optimization Variable

Instead of optimizing over the nonnegative factors **D** and **H**, we introduce the variables ***δ*** and ***η***, which are related to **D** and **H** by

(17) $$\begin{array}{cc} D & =g_{d}(\delta )={\text{vec}}^{-1}\left(f_{d}^{-1}(C_{d}^{⊤}\delta )\right),\\ H & =g_{h}(\eta )={\text{vec}}^{-1}\left(f_{h}^{-1}(C_{h}^{⊤}\eta )\right), \end{array}$$

where the vec^−1^(⋅) function maps its vector input into a matrix of appropriate size. The matrices **C**_*d*_ and **C**_*h*_ are the matrix square roots (Cholesky decompositions) of the covariance matrices **Σ**_*d*_ and **Σ**_*h*_, such that ***δ*** and ***η*** are standard i.i.d. Gaussian.

This change of variable serves two purposes. First of all, we found that optimizing over the transformed variables was faster, more robust, and less prone to getting stuck in local minima. Second, the transformed variables are not constrained to be nonnegative, which allows us to use existing unconstrained optimization methods to compute their MAP estimate.

The prior distribution of the transformed variable ***η*** is

(18) $$p_{\eta }(\eta )=p_{H}(g_{h}(\eta ))\;|\;J(g_{h}(\eta ))\;|=\frac{1}{{\left(2\pi \right)}^{LM/2}}\exp\left(-\frac{1}{2}\eta^{⊤}\eta \right),$$

and the negative log prior is

(19) $${ℒ}_{\eta }(\eta )\propto \frac{1}{2}\eta^{⊤}\eta .$$

To compute the MAP estimate of the transformed variables, we must combine this expression for the prior (and a similar expression for the prior of ***δ***) with a likelihood function, in which the same change of variable is made

(20) $${ℒ}_{\delta ,\eta |X}(\delta ,\eta )={ℒ}_{X|D,H}(g_{d}(\delta ),g_{h}(\eta ))+\frac{1}{2}\delta^{⊤}\delta +\frac{1}{2}\eta^{⊤}\eta .$$

Then, the MAP solution can be found by optimizing over ***δ*** and ***η*** as

(21) $$\{\delta_{\text{MAP}},\eta_{\text{MAP}}\}=\underset{\delta ,\eta }{\arg\min}{ℒ}_{\delta ,\eta |X}(\delta ,\eta ),$$

and, finally, estimates of **D** and **H** can be computed using (17).

**Example 3 (least squares nonnegative matrix factorization with Gaussian process priors (GPP-NMF)).**

If we use the least squares likelihood in (4), the posterior distribution in (20) is given by

(22) $${ℒ}_{\delta ,\eta |X}^{\text{LS}\text{‐}\text{GPP}}(\delta ,\eta )=\frac{1}{2}\left(\sigma_{N}^{-2}∥X-g_{d}(\delta )g_{h}(\eta ){∥}_{F}^{2}+\delta^{⊤}\delta +\eta^{⊤}\eta \right).$$

The MAP estimate of ***δ*** and ***η*** is found by minimizing this expression, for which the derivative is useful

(23) $$\nabla_{\eta }{ℒ}_{\delta ,\eta |X}^{\text{LS}\text{‐GPP}}(\delta ,\eta )=\sigma_{N}^{-2}(\text{vec}\left(g_{d}{\left(\delta \right)}^{⊤}(g_{d}(\delta )g_{h}(\eta )-X)\right){\;⊙\;{\left(f_{h}^{-1}\right)}^{\prime }(C_{h}^{⊤}\eta ))}^{⊤}C_{h}+\eta ,$$

where ⊙ denotes the Hadamard (element-wise) product. The derivative with respect to ***δ*** is similar due to the symmetry of the NMF problem.

### 2.5. Link Function

Any strictly increasing link function that maps the nonnegative reals to the real line can be used in the proposed framework; however, in order to have a better probabilistic interpretation of the prior distribution, we propose a simple principle for choosing the link function. We choose the link function such that *f_h_* ^−1^ maps the marginal distribution of the elements of the underlying Gaussian process vector **h** into a specifically chosen marginal distribution of the elements of **H**. Such an inverse function can be found as *f_h_* ^−1^(**h**_*i*_) = *P_H_* ^−1^(*P_h_*(**h**_*i*_)), where *P*(⋅) denotes the marginal cumulative distribution functions (CDFs).

Since the marginals of a Gaussian process are Gaussian, *P_h_*(**h**_*i*_) is the Gaussian (CDF), and, using (13), the inverse link function is given by

(24) $$f_{h}^{-1}(h_{i})={\text{P}}_{H}^{-1}\left(\frac{1}{2}+\frac{1}{2}\Phi \left(\frac{h_{i}}{\sqrt{2}\sigma_{i}}\right)\right),$$

where *σ_i_* ^2^ is the *i*th diagonal element of **Σ**_*h*_ and Φ(⋅) is the error function.

**Example 4 (exponential-to-Gaussian link function).**

If we choose to have exponential marginals in **H**, as in NNSC described in Example 2, we select *P_H_* as the exponential CDF. The inverse link function is then

(25) $$f_{h}^{-1}(h_{i})=-\frac{1}{\lambda }\log\left(\frac{1}{2}-\frac{1}{2}\Phi \left(\frac{h_{i}}{\sqrt{2}\sigma_{i}}\right)\right),$$

where *λ* is an inverse scale parameter. The derivative of the inverse link function, which is needed for the parameter estimation, is given by

(26) $${\left(f_{h}^{-1}\right)}^{\prime }(h_{i})=\frac{1}{\sqrt{2\pi }\sigma_{i}\lambda }\text{exp}\left(\lambda f_{h}^{-1}(h_{i})-\frac{h_{i}^{2}}{2\sigma_{i}^{2}}\right).$$

**Example 5 (rectified-Gaussian-to-Gaussian link function).**

Another interesting nonnegative distribution is the rectified Gaussian given by

(27) $$p(x)=\{\begin{array}{ll} \frac{2}{\sqrt{2\pi }s}\exp\left(-\frac{x^{2}}{2s^{2}}\right), & \;x\geq 0,\\ 0, & \;x<0. \end{array}$$

Using this pdf in (24), the inverse link function is

(28) $$f_{h}^{-1}(h_{i})=\sqrt{2}s\Phi^{-1}\left(\frac{1}{2}+\frac{1}{2}\Phi \left(\frac{h_{i}}{\sqrt{2}\sigma_{i}}\right)\right),$$

and the derivative of the inverse link function is

(29) $${\left(f_{h}^{-1}\right)}^{\prime }(h_{i})=\frac{s}{2\sigma_{i}}\exp\left(\frac{f_{h}^{-1}{\left(h_{i}\right)}^{2}}{2s^{2}}-\frac{h_{i}^{2}}{2\sigma_{i}^{2}}\right).$$

### 2.6. Summary of the GPP-NMF Method

The GPP-NMF method can be summarized in the following steps.

- Choose a suitable negative log-likelihood function *ℒ*_*X*∣*D*,*H*_(**D**,**H**) based on knowledge of the distribution of the data or the residual.
- For each of the nonnegative factors **D** and **H**, choose suitable link and covariance functions according to your prior beliefs. If necessary, draw samples from the prior distribution to examine its properties.
- Compute the MAP estimate of ***δ*** and ***η*** by (21) using any suitable unconstrained optimization algorithm.
- Compute **D** and **H** using (17).

Our Matlab implemention of the GPP-NMF method is available online [23].

## 3. Experimental Results

We will demonstrate the proposed method on two examples, first a toy example, and second an example taken from the chemical shift brain imaging literature.

In our experiments, we use the least squares GPP-NMF described in Example 3 and the link functions described in Examples 4-5. The specific optimization method used to compute the GPP-NMF MAP estimate is not the topic of this paper, and any unconstrained optimization algorithm could in principle be used. In our experiments, we used a simple gradient descent with line search to perform a total of 1000 alternating updates of ***δ*** and ***η***, after which the algorithm had converged. For details of the implementation, see the accompanying Matlab code [23].

### 3.1. Toy Example

**Figure 1:**
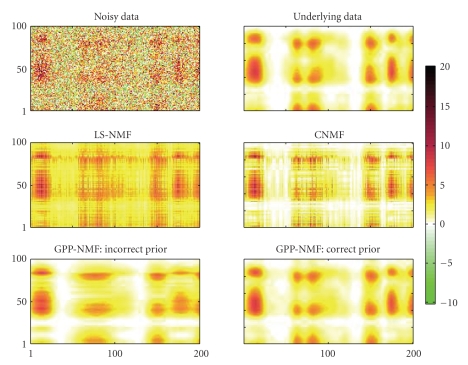
Toy example data matrix (upper left), underlying noise-free nonnegative data (upper right), and estimates using the four methods described in the text. The data has a fairly large amount of noise, and the underlying nonnegative factors are smooth in both directions. The LS-NMF and CNMF decompositions are nonsmooth since these methods are not model of correlations in the factors. The GPP-NMF, which uses a smooth prior, finds a smooth solution. When using the correct prior, the soulution is very close to the true underlying data.

We generated a 100 × 200 data matrix, **Y**, by taking a random sample from the GPP-NMF model with two factors. We chose the generating covariance function for both ***δ*** and ***η*** as a Gaussian radial basis function (RBF)

(30) $$\phi (i,j)=\exp\left(-\frac{{\left(i-j\right)}^{2}}{\beta^{2}}\right),$$

where *i* and *j* are two sample indices, and the length-scale parameter, which determines the smoothness of the factors, was *β* ^2^ = 100. We set the covariance between the two factors to zero, such that the factors were uncorrelated. For the matrix **D**, we used the rectified-Gaussian-to-Gaussian link function with *s* = 1; and for **H**, we used the exponential-to-Gaussian link function with *λ* = 1. Finally, we added independent Gaussian noise with variance *σ_N_* ^2^ = 25, which resulted in a signal-to-noise ratio of approximately −7 dB. The generated data matrix is shown in Figure 1.

We then decomposed the generated data matrix using the following four different methods:

- ***LS-NMF*:** standard least squares NMF [8]. This algorithm does not allow negative data points, so these were set to zero in the experiment.
- ***CNMF*:** constrained NMF [6, 7], which is a least squares NMF algorithm that allows negative observations.
- ***GPP-NMF*: *correct prior*:** the proposed method with correct link functions, covariance matrix, and parameter values.
- ***GPP-NMF*: *incorrect prior*:** to illustrate the sensitivity of the method to prior assumptions, we evaluated the proposed method with incorrect prior assumptions: we switched the link functions, such that for **D** we used the exponential-to-Gaussian, and for **H** we used the rectified-Gaussian-to-Gaussian. We used an RBF covariance function with *β* ^2^ = 10 and *β* ^2^ = 1000 for **D** and **H**, respectively.

The results of the decompositions are shown as reconstructed data matrices in Figure 1. All four methods find solutions that visually appear to fit the underlying data. Both LS-NMF and CNMF find nonsmooth solutions, whereas the two GPP-NMF results are smooth in accordance with the priors. In the GPP-NMF with incorrect prior, the dark areas (high-pixel intensities) appear too wide in the first axis direction and too narrow in the section axis direction, due to the incorrect setting of the covariance function. The GPP-NMF with correct prior is visually almost equal to the true underlying data.

**Figure 2:**
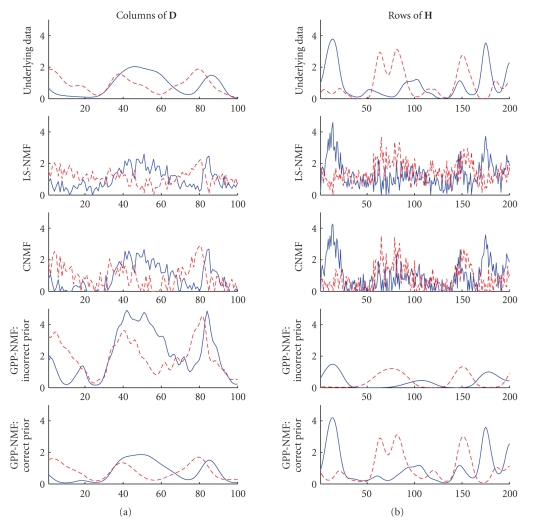
Underlying nonnegative factors in the toy example: columns of **D** (left) and rows of **H** (right). The factors found by the LS-NMF and the CNMF algorithms are noisy, whereas the factors found by the GPP-NMF method are smooth. When using the correct prior, the factors found are very similar to the true factors.

Plots of the estimated factors are show in Figure 2. The factors estimated by the LS-NMF and the CNMF methods appear noisy and are nonsmooth, whereas the factors estimated by the GPP-NMF are smooth. The factors estimated by the LS-NMF method have a positive bias, because of the truncation of negative data. The GPP-NMF with incorrect prior has too many local extrema in the **D** factor and too few in the **H** factor due to the incorrect covariance functions. There are only minor difference between the result of the GPP-NMF with the correct prior and the underlying factors.

**Figure 3:**
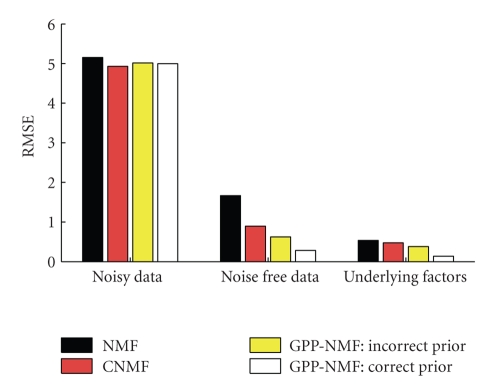
Toy example: root mean squared error (RMSE) with respect to the noisy data, the underlying noise-free data, and the true underlying nonnegative factors. The CNMF solution fits the noisy data slightly better, but the GPP-NMF solution fits the underlying data much better.

Measures of root mean squared error (RMSE) of the four decompositions are given in Figure 3. All four methods fit the noisy data almost equally well. (Note that, due to the additive noise with variance 25, a perfect fit to the underlying factors would result in an RMSE of 5 with respect to the noisy data.) The LS-NMF fits the data worst due to the truncation of negative data points, and the CNMF fits the data best, due to overfitting. With respect to the noise-free data and the underlying factors, the RMSE is worst for the LS-NMF and best for the GPP-NMF with correct prior. The GPP-NMF with incorrect prior is better than both LS-NMF and CNMF in this case. This shows that in this situation it is better to use a prior which is not perfectly correct, compared to using no prior as in the LS-NMF and CNMF methods, (which corresponds to a flat prior over the nonnegative reals and no correlations).

### 3.2. Chemical Shift Srain Imaging Example

**Figure 4:**
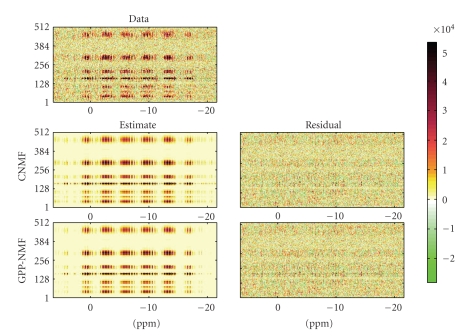
Brain imaging data matrix (top) along with the estimated decomposition and residual for the CNMF (middle) and GPP-NMF (bottom) methods. In this view, the results of the two decompositions are very similar, the data appears to be modeled equally well and the residuals are similar in magnitude.

Next, we demonstrate the GPP-NMF method on ^1^H decoupled ^31^P chemical shift imaging data of the human brain. We use the data set from Ochs et al. [24], which has also been analyzed by Sajda et al. [6, 7]. The data set, which is shown in Figure 4, consists of 512 spectra measured on an 8 × 8 × 8 grid in the brain.

Ochs et al. [24] use PCA to determine that the data set is adequately described by two sources (which correspond to brain and muscle tissue). They propose a bilinear Bayesian approach, in which they use a smooth prior over the constituent spectra, and force to zero the amplitude of the spectral shape corresponding to muscle tissue at 12 positions deep inside the head. Their approach produces physically plausible results, but it is computationally very expensive and takes several hours to compute.

Sajda et al. [6, 7] propose an NMF approach that is reported also to produce physically plausible results. Their method is several orders of magnitude faster, taking less than a second to compute. The disadvantage of the method of Sajda et al. compared to the Bayesian approach of Ochs et al. is that it provides no mechanism for using prior knowledge to improve the solution.

**Figure 5:**
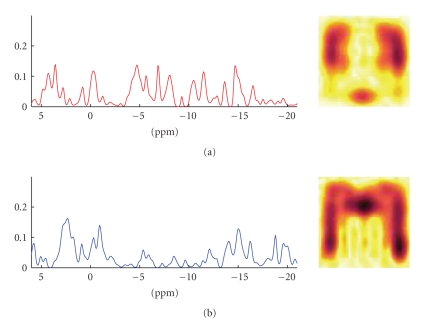
Brain imaging data: random draw from the prior distribution with the parameters set as described in the text. The prior distribution of the constituent spectra (left) is exponential and smooth, and the spatial distribution (right) in the brain is exponential, smooth, and has a left-to-right symmetry.

The GPP-NMF approach we propose in this paper bridges the gap between the two previous approaches, in the sense that it is a relatively fast NMF approach, in which priors over the factors can be specified. These priors are specified by the choice of the link and covariance functions. We used prior predictive sampling to find reasonable settings of the the function parameters: we drew random samples from the prior distribution and examined properties of the factors and reconstructed data. We then manually adjusted the parameters of the prior to match our prior beliefs. An example of a random draw from the prior distribution is shown in Figure 5, with the parameters set as described below.

We assumed that the factors are uncorrelated, so the covariance between factors is zero. We used a Gaussian RBF covariance function for the constituent spectra, with a length scale of *β* = 0.3 parts per million (ppm), and we used the exponential-to-Gaussian link function with *λ_d_* = 1. This gave a prior for the spectra that is sparse with narrow smooth peaks. For the amplitude at the 512 voxels in the head, we used a Gaussian RBF covariance function on the 3D voxel indices, with length scale *β* = 2. Furthermore, we centered the left-to-right coordinate axis in the middle of the brain, and computed the RBF kernel on the absolute value of this coordinate, so that a left-to-right symmetry was introduced in the prior distribution. Again, we used the exponential-to-Gaussian link function, and we chose *λ_h_* = 2 ⋅ 10^−4^ to match the overall magnitude of the data. This gave a prior for the amplitude distribution that is sparse, smooth, and symmetric. The noise variance was set to *σ_N_* ^2^ = 10^8^ which corresponds to the noise level in the data set.

**Figure 6:**
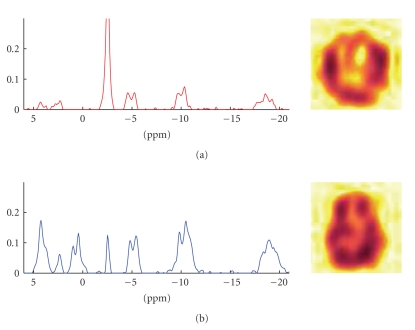
CNMF decomposition result. The recovered spectra are physically plausible, and the spatial distribution in the brain for the muscle (top) and brain (bottom) tissue is somewhat separated. Muscle tissue is primarily found near the edge of the skull, whereas brain tissue is primarily found at the inside of the head.

**Figure 7:**
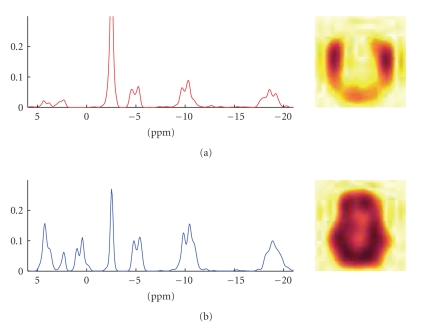
GPP-NMF decomposition result. The recovered spectra are very similar to the spectra found by the CNMF method, but they are slightly more smooth. The spatial distribution in the brain is highly separated between brain and muscle tissue, and it is more symmetric than the CNMF solution.

We then decomposed the data set using the proposed GPP-NMF algorithm and, for comparison, reproduced the results of Sajda et al. [7] using their CNMF method. The results of the experiments are shown in Figure 4. An example of a random draw from the prior distribution is shown in Figure 5. The results of the CNMF is shown in Figure 6, and the results of the GPP-NMF is shown in Figure 7. The figures show the constituent spectra and the fifth axial slice of the spatial distribution of the spectra. The 8 × 8 spatial distributions are smoothed in the illustration, similar to the way the results are visualized in the literature.

The results show that both methods give physically plausible results. The main difference is that the spatial distribution of the spectra corresponding to muscle and brain tissue is much more separated in the GPP-NMF result, which is due to the exponential, smooth, and symmetric prior distribution. By including prior information, we obtain a solution, where the factor corresponding to muscle tissue is clearly located on the edge of the skull.

## 4. Conclusions

We have introduced a general method for exploiting prior knowledge in nonnegative matrix factorization, based on Gaussian process priors, linked to the nonnegative factors by a link function. The method can be combined with any existing NMF cost function that has a probabilistic interpretation, and any existing unconstrained optimization algorithm can be used to compute the maximum a posteriori estimate.

Experiments on toy data show that with a suitable selection of the prior distribution of the nonnegative factors, the GPP-NMF method gives much better results in terms of estimating the true underlying factors, both when compared to traditional NMF and CNMF.

Experiments on chemical shift brain imaging data show that the GPP-NMF method can be successfully used to include prior knowledge of the spectral and spatial distribution, resulting in better spatial separation between spectra corresponding to muscle and brain tissue.
